# Arylcyanomethylenequinone Oximes: An Overview of Synthesis, Chemical Transformations, and Biological Activity

**DOI:** 10.3390/molecules28135229

**Published:** 2023-07-05

**Authors:** Karolina Kula, Roman Nagatsky, Mikołaj Sadowski, Yevheniia Siumka, Oleg M. Demchuk

**Affiliations:** 1 Department of Organic Chemistry and Technology, Cracow University of Technology, Warszawska 24, 31-155 Krakow, Poland; 2 Institute for Advanced Training of Pharmacy Specialists, National University of Pharmacy, 17 Zahysnynykiv Ukrainy sq., 61001 Kharkiv, Ukraine; 3 Faculty of Medicine, The John Paul II Catholic University of Lublin, Konstantynow 1J, 20-708 Lublin, Poland

**Keywords:** quinonemethide, arylcyanomethylenequinone oximes, quinone, chemical transformation, biological activity

## Abstract

Quinone methides are a class of biologically active compounds that can be used in medicine as antibacterial, antifungal, antiviral, antioxidant, and anti-inflammatory agents. In addition, quinone methides have the potential to be used as pesticides, dyes, and additives for rubber and plastics. In this paper, we discuss a subclass of quinone methides: methylenequinone oximes. Although the first representatives of the subgroup were synthesized in the distant past, they still need to be additionally studied, while their chemistry, biological properties, and perspective of practical applications require to be comprehensively summarised. Based on the analysis of the literature, it can be concluded that methylenequinone oximes exhibit a diversified profile of properties and outstanding potential as new drug candidates and reagents in organic synthesis, both of electrophilic and nucleophilic nature, worthy of wide-ranging further research.

## 1. Introduction

Quinones, and especially benzoquinones, play an important role in the metabolism of all living cells, since they have the ability to act as mediators for both electron [1] and proton transfers [2,3]. Worth noting is also the fact that redox reactions mediated by benzoquinones may lead to the creation of reactive oxygen species, causing cellular damage [4,5]. At the same time, particularly substituted quinones can behave as antioxidants, protecting healthy cells [6]. Such a widespread pattern of activity of quinones makes some of them efficient antibiotics [7,8] and vitamins [9]. The shortcomings of currently used drugs dictate the urgent and growing need to discover the new classes of medicines and some quinonemethide derivatives may be useful to fill that gap.

Quinone methides are the derivatives of quinones in which one of the oxygen atoms is replaced with carbon. The chemistry of quinone methides is less developed and their biological properties are less explored. These compounds have a conjugated system, making them favourable for nucleophilic addition via an exocyclic double bond. It is worth noting that quinone methides are of more polar nature, compared to quinones; therefore, they are considered to have higher reactivity [10]. Most quinone methides have pronounced biological activity. Among biologically active substances containing quinone methide backbone, many can be found with antitumor [11,12,13], antibacterial [14,15,16], antifungal [17], DNA alkylating and cross-linking agent [18], antioxidant [19,20], and anti-inflammatory [19,21] properties. Moreover, the main pharmacological mechanism of action of some other anticancer phenolic drugs is based on active quinone methides that are metabolically formed in living cells [22].

A subgroup of quinone methides are their oximes. They represent a rather attractive group of organic compounds, since their natural and synthetic derivatives are characterized by a diverse range of biological properties, therefore making them an interesting object for scientists working in the fields of organic, pharmaceutical, and medicinal chemistry, as well as chemistry of natural compounds. Many quinone methide oximes have already found their application in industry, including pharmaceutical and medical sectors: namely, as components of photoresists [23,24], colour filters [25], additives for rubber [26,27] and plastic products [28,29,30,31], and as herbicides [32]. The quinone methide oximes were found to be intermediates in the organic synthesis of already known biologically active substances [33,34] and other important products [35,36]. It has also been reported that oximes of quinone methides can be used as antidotes [37,38], disinfectants [39], or to obtain prodrugs [40]. What is more interesting is that various quinone methide oxime derivatives reportedly possess antifungal [41,42] and antitumor [39,43] activities. The presence of a conjugated system in the molecules of quinone methide oximes allows for them to be used in the manufacturing of various dyes as well [44].

**Figure 1:**
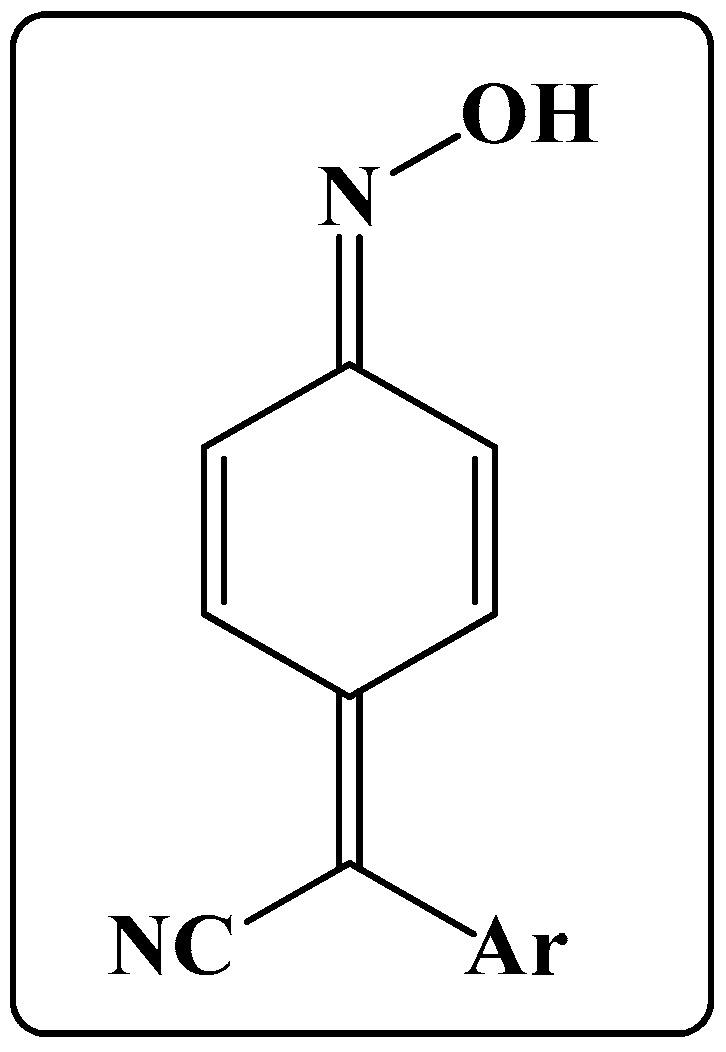
The structure of arylcyanomethylenequinone oximes studied in this work.

Based on the analysis of the literature, it can be concluded that synthetic and pharmaceutical chemistry, as well as the biological properties of quinone methide oxime derivatives are fairly diverse. The objective of this review is to shed some light on a subgroup of quinone methide oximes derivatives, namely, arylcyanomethylenequinone oximes (Figure 1). Throughout the review, the synthesis, methods of chemical modification, and biological activity of the subgroup are highlighted.

Although the first representatives of these compounds were synthesized in the distant past [45], they are still insufficiently studied. To the best knowledge of the authors of this review, there is an absence of a professional literature review discussing arylcyanomethylenequinone oximes in detail. Several papers dedicated to the specific types of quinone methides put their focus on the generation of quinone methide core and its reactivity [46], on the application of quinone methides in asymmetric organocatalysis [47], on their biological properties [48,49], and potential application, e.g., in the fluorescent sensing of enzymatic activity [50]. The polycyclic friedelane-type triterpenoid quinone methides [51] and pyridinium quinone methide derivatives [52] are also known to be biologically active.

Due to the previously specified deficits in the literature review of the topic, we hereby decided to systemize the current knowledge of arylcyanomethylenequinone oximes covering the period between the earliest publications from 1960 until the contemporary publication from 2021. The availability and easiness of the synthesis make the discussed compounds even more favourable for potential usage in industry, medicine, and as precursors for organic synthesis. What is more important, the presence of two valuable moieties, namely, cyano- and oxime groups as well as dual electrophilic–nucleophilic nature of the arylcyanomethylenequinone oximes, render them very attractive objects for further transformations [24,25,32,42,53,54,55,56,57,58,59]. However, what makes (hetero) arylcyanomethylenequinone oximes, and their derivatives, interesting research subjects, especially in the field of medicine, is their biological activity. Among others, the most promising is potential usage of arylcyanomethylenequinone oximes as antitumor [60], antiparasitic [61], antifungal [41,62], anti-allergic [63], and anti-inflammatory [64] agents. In some works, there is also information that arylcyanomethylenequinone oximes can be used as plant protection herbicides [32,65], fungicides [66] and pesticides [67]. In conjunction with the previous information, we hope that the summarized description of the synthesis, chemical transformations and biological properties of arylcyanomethylenequinone oximes will turn out to be useful to the chemists researching new aspects of the medicinal chemistry.

## 2. Synthesis of Arylcyanomethylenequinone Oximes and Some of Their Derivatives

### 2.1. The Condensation of (Hetero) Arylacetonitriles with Nitro (Hetero) Arenes

There are not many methods applicable to the synthesis of both arylcyanomethylenequinone oximes and other quinone methide oxime derivatives. The analysis of the literature data showed that one of the best approaches for the construction of their backbone is the condensation of benzyl cyanides with 4-unsubstituted nitroarenes or nitroheteroarenes [60], which is a simple way of synthesis of various derivatives of such oximes.

**Scheme 1:**
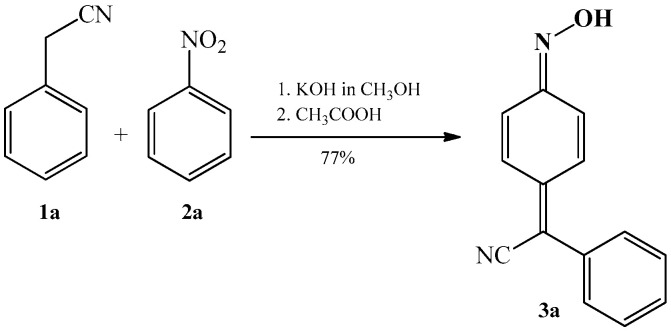
Condensation reaction of benzyl cyanide **1a** with nitrobenzene **2a** that produces 4-(phenylcyanomethylene)-cyclohexa-2,5-dien-1-one oxime **3a**.

The synthesis of phenylcyanomethylenequinone oxime was first described by Davis et al. [45] in 1960. The authors stated that when benzyl cyanide **1a** and nitrobenzene **2a** are added to a warm alcoholic solution of potassium hydroxide, the reaction mixture becomes dark red and a like-coloured solid soon precipitates. The solid dissolves in water, and upon acidification with acetic acid solution, a new yellow–orange solid precipitates with a yield of 77%; the latter solid is 4-(phenylcyanomethylene)-cyclohexa-2,5-dien-1-one oxime **3a** (Scheme 1).

**Figure 2:**
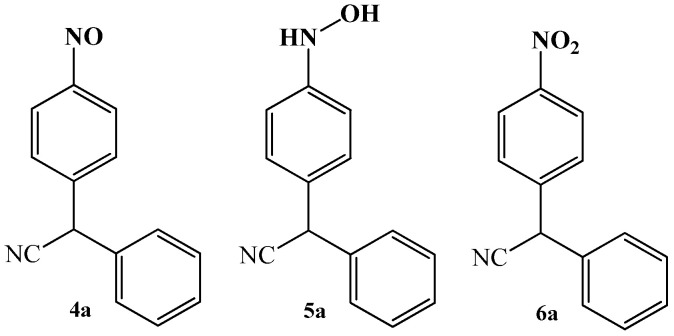
Structures of other potential reaction products of condensation reaction between benzyl cyanide **1a** and nitrobenzene **2a**.

Simultaneously, in that work, the authors also reported that no evidence for the other possible reaction products, except for product **3a**, were obtained. Attempts to isolate possible structures **4a**, **5a**, **6a** were unsuccessful (Figure 2).

**Scheme 2:**
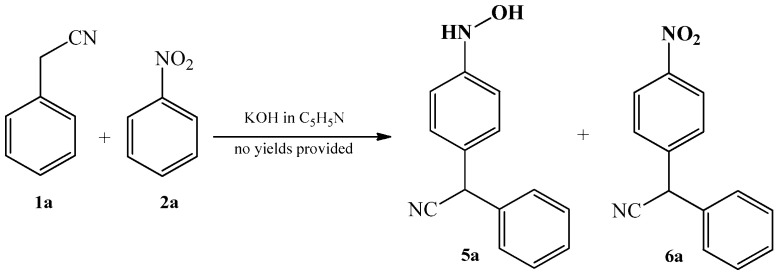
Condensation reaction of benzyl cyanide **1a** with nitrobenzene **2a** to produce analogues **5a** and **6a** of oxime **3a**.

On the other hand, they have noted that in the reaction between benzyl cyanide **1a** and nitrobenzene **2a,** it is possible to obtain other products when the methanol solvent was replaced with pyridine: the mixture of products **5a** and **6a** (Scheme 2) was obtained then.

**Scheme 3:**
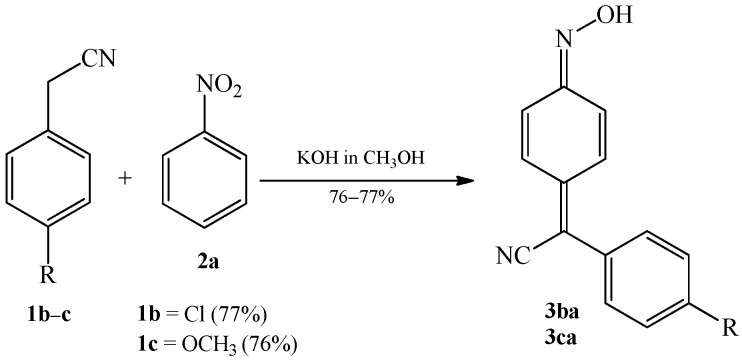
Condensation reaction of 4-substituted benzyl cyanides **1b**–**c** with nitrobenzene **2a** to produce quinone oximes **3ba** and **3ca**.

**Scheme 4:**
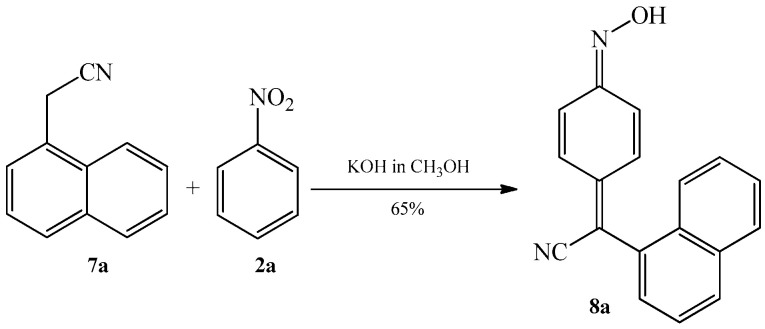
Condensation reaction of α-naphthylacetonitrile **7a** with nitrobenzene **2a** to produce quinone oximes **8a**.

In the same paper the authors have also shown, for the first time, that nitrobenzene **2a** can react with 4-substituted benzyl cyanides, such as 4-chlorobenzyl cyanide **1b** and 4-methoxybenzyl cyanide **1c**, as well as with α-naphthylacetonitrile **7a**. Reactions took place in a methanolic solution of potassium hydroxide, forming corresponding quinone oximes **3ba**, **3ca** (Scheme 3), and **8a** (Scheme 4).

**Scheme 5:**
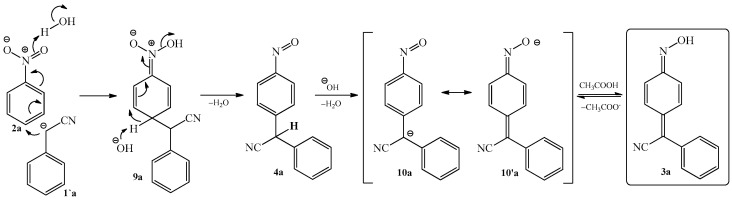
Mechanism of condensation reaction of nitrobenzene **1a** with benzyl cyanide **2a** to produce 4-(phenylcyanomethylene)-cyclohexa-2,5-dien-1-one oxime **3a**.

The mechanism of condensation reaction between benzyl cyanide **1a** and nitrobenzene **2a** was described on Scheme 5. In the first step, nitrobenzene **2a** undergoes nucleophilic attack of the benzyl cyanide anion **1’a** at the 4-position, with subsequent addition of a proton to the oxygen of the nitro group to form intermediate **9a**, which can also exist as a potassium salt. Further elimination of a water molecule leads to the formation of the nitroso derivative **4a**. In turn, compound **4a** rapidly transforms into an anion, possessing two alternative forms **10a** and **10’a**. Finally, acidification of the potassium salt **10’a** leads to the formation of phenylcyanomethylenequinone oxime **3a**.

In 1962, Lichtenberger and Weiss [68] reproduced the condensation reaction between nitrobenzene **1a** and benzyl cyanide **2a**, proposed by Davis et al. [45]. The authors reported that they had obtained 4-(phenylcyanomethylene)-cyclohexa-2,5-dien-1-one oxime **3a** as the only reaction product, thus confirming previous studies.

**Scheme 6:**
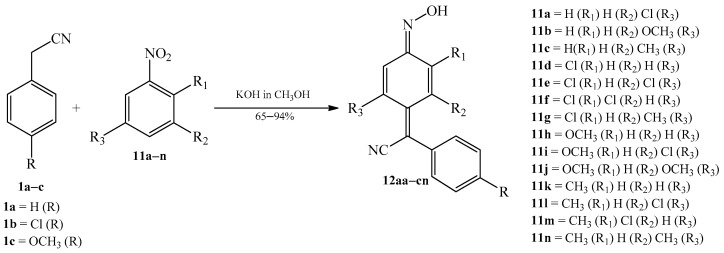
Condensation reaction of 4-substituted benzyl cyanides **1a**–**c** with 4-unsubstituted analogues of nitrobenzene **11a**–**n** to produce quinone oximes **12aa**–**cn**.

In 1961, Davis et al., continuing the study of the course of the condensation of various 4-substituted benzyl cyanides **1a**–**c** with 4-unsubstituted nitroarenes **11a**–**n** (Scheme 6), published the work [69] in which detailed preparations, by the previously shown condensation method, were described. As a result, 34 new arylcyanomethylenequinone oxime analogues **12aa**–**cn** were synthesized (Table 1). The reactions were realized in warm alcoholic solution of potassium hydroxide. This work showed what a large collection of compounds can be synthesized by varying virtually only four substituents (-H, -Cl, -OCH_3_ and -CH_3_).

**Scheme 7:**
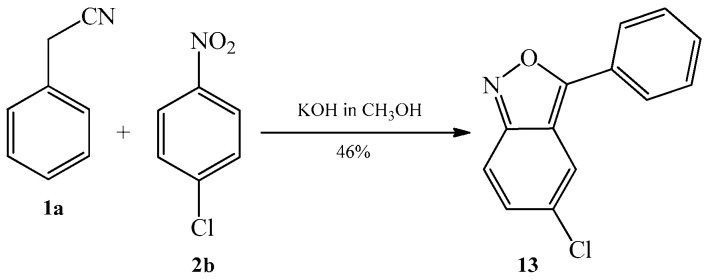
Condensation reaction of benzyl cyanide **1a** with 4-chloronitrobenzene **2b** to produce 3-phenyl-5-chloroanthranil **13**.

In 1964, Davis [70] patented another example of condensation of benzyl cyanide **1a** with 4-chloronitrobenzene **2b**. The author described that in a course of reaction, the expected arylcyanomethylenequinone oxime is not created but an analogue of isoxazole **13** is formed as the only product (Scheme 7). The reaction was carried out in warm alcoholic solution of potassium hydroxide.

**Scheme 8:**
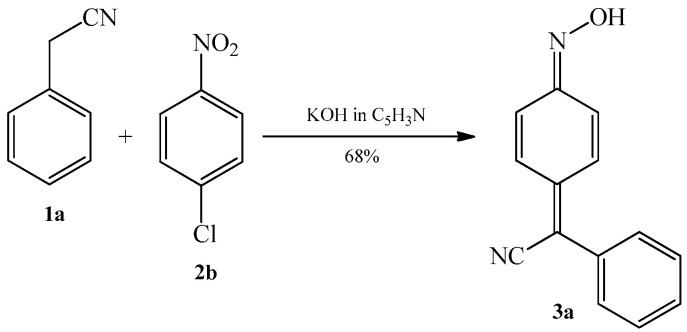
Condensation reaction of benzyl cyanide **1a** with 4-chloronitrobenzene **2b** to produce 4-(phenylcyanomethylene)-cyclohexa-2,5-dien-1-one oxime **3a**.

In the same patent, Davis reported that in the reaction between benzyl cyanide **1a** and 4-chloronitrobenzene **2b,** when the solvent is changed from methanol to pyridine, the 4-chloro substituent is not retained in the product. In turn, 4-(phenylcyanomethylene)-cyclohexa-2,5-dien-1-one oxime **3a** is formed (Scheme 8). This variation of the reaction, in contrast to the previous examples, allows to use 4-substituted nitroarenes in the synthesis of arylcyanomethylenequinone oximes.

**Scheme 9:**
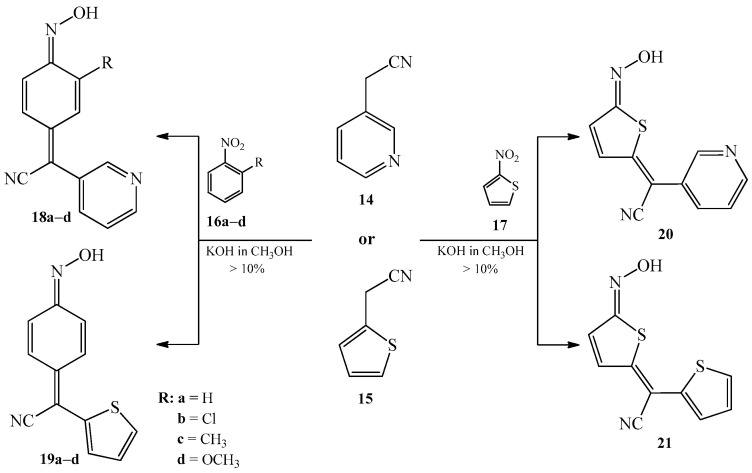
Condensation reaction of 3-pyridylacetonitrile **14** or 2-thienylacetonitrile **15** with 2-substituted nitrobenzene analogues **16a**–**c** and 2-nitrothiophene **17** to produce oximes **18**–**21**.

In 1965, Marey et al. [71] proposed the use of heteroaromatic analogues of benzyl cyanide **2a** such as 3-pyridylacetonitrile **14** and 2-thienylacetonitrile **15** and (hetero)aromatic nitro compounds like 2-substituted nitrobenzene **16a**–**c** and 2-nitrothiophene **17** in the condensation reaction. Reactions took place in warm methanolic solution of potassium hydroxide, forming corresponding heterocyclic analogues of arylcyanomethylenequinone oximes **18**–**21** (Scheme 9). The authors mentioned that yields of all reactions were >10%.

**Scheme 10:**
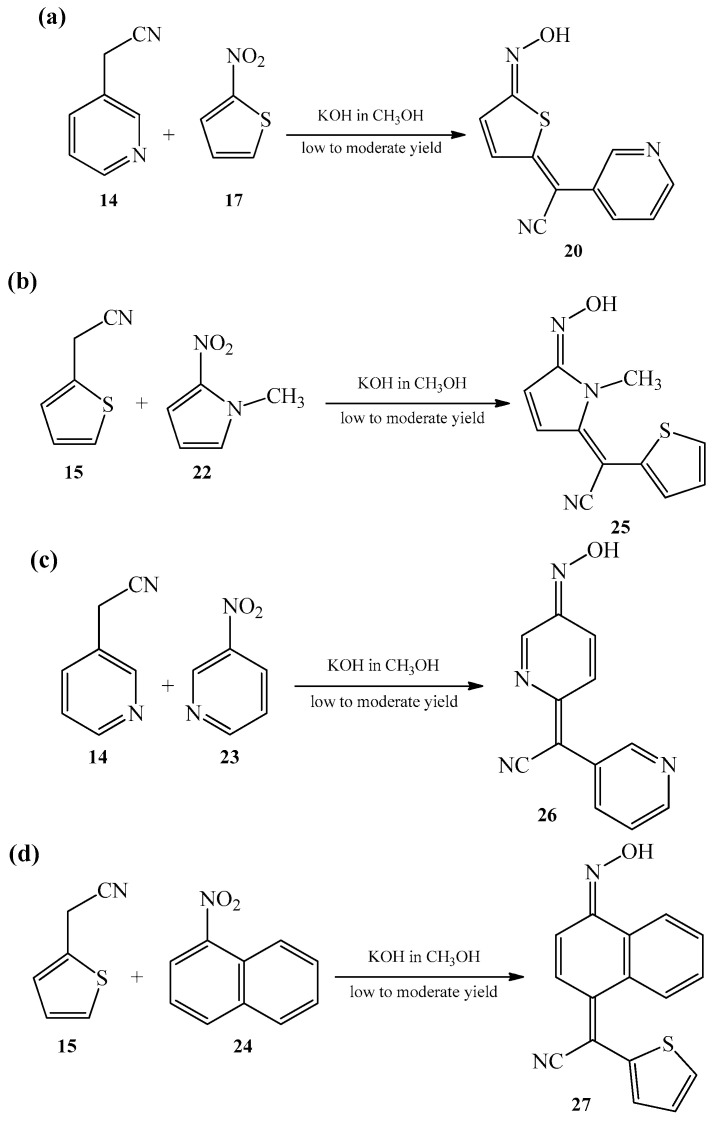
Condensation reaction between nitriles **14** and **15** and (hetero)aromatic nitro compounds **17, 22**–**24** forming heterocyclic analogues **20**, **25** (**a**,**b**), heteroatom containing **26** (**c**), and bi(carbo)cyclic **27** (**d**) heteroarylcyanomethylenequinone oximes.

Continuing the study of synthesis of such heterocyclic oximes, in 1968, Fournari and Marey [72] reported on the study of reactions between heteroarylacetonitriles **14** and **15** with (hetero)aromatic nitro compounds **17** and **22**–**24**. Reactions took place in a way analogous to the previous one, namely, deploying methanolic solution of potassium hydroxide, forming corresponding oximes **20** and **25**–**27** (Scheme 10a–d), thereby increasing the range of known oxime derivatives. The compounds were formed in low to moderate yields.

**Scheme 11:**
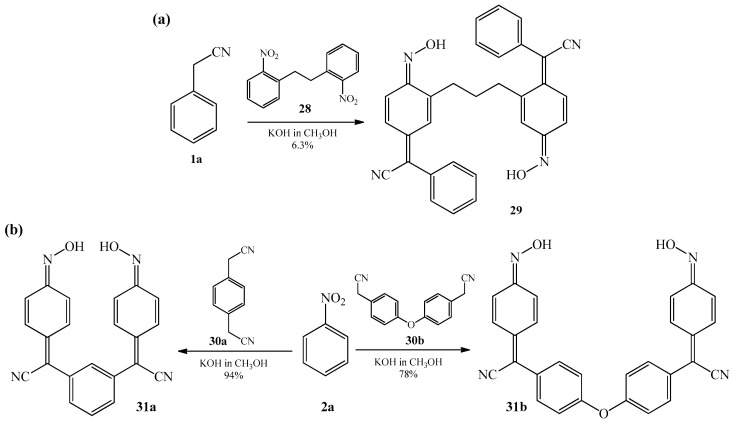
(**a**) Condensation reaction of benzyl cyanide **1a** with 2,2′-dinitrobibenzyl **28**; (**b**) Condensation reaction of nitrobenzene **2a** with 1,4-bis(cyanomethyl)benzene **30a** and 1,4-bis (cyanomethyl) diphenyl ether **30b**.

In the same year, Mitchell in two patents [73,74] described synthesis of derivatives of bis (phenylacetonitrile) oxime **29** and **31a**–**b** in a reaction of condensation of benzyl cyanide **1a** with 2,2′-dinitrobibenzyl **28** (Scheme 11a) as well as 1,4-bis (cyanomethyl) benzene **30a** with nitrobenzene **2a** (Scheme 11b) and 1,4-bis (cyanomethyl) diphenyl ether **30b**, also, with nitrobenzene **2a** (Scheme 11b). All reactions were realized in warm alcoholic solution of potassium hydroxide.

In the 1970s, Takahashi et al. began studying the possibility of deploying catalysts and their effect on the course of the condensation reactions. Their research led to forming 4-(phenylcyanomethylene)-cyclohexa-2,5-dien-1-one oxime **3a** in a reaction of benzyl cyanide **1a** with nitrobenzene **2a** mediated by base catalyst [75]. In 1984, Arseniyadis et al. [76] described the mechanisms of addition and substitution reactions of nitrile-stabilized carbanions, including the mechanism of the formation of 4-quinone methide oximes. Later, Freyne and Raeymaekers in 1991 [77], Freyne et al. in 1996 [78], Makosza and Wróbel in 1996 [79], Wróbel in 2000 [80], Yamato et al. in 2000 [24], Suwiński et al. in 2003 [81], Orlov et al. in 2007 [82], Konovalova et al. in 2008 [83], Orlov et al. in 2009 [84], Buehler et al. in 2010 [85], Orlov et al. in 2010 [86], and Hong et al. in 2016 [60] mentioned the synthesis of 4-(phenylcyanomethylene)-cyclohexa-2,5-dien-1-one oxime **3a** by the already presented condensation of benzyl cyanide **1a** with 4-unsubstituted nitrobenzenes **11a**–**n** with attempts to optimize the conditions of these reactions via the use of catalysts, and changes of solvents, reaction times and temperature regimes, among other modifications.

**Scheme 12:**
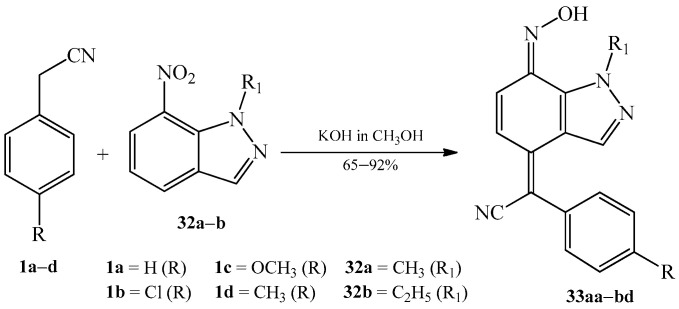
Condensation reaction of various 4-substituted benzyl cyanides **1a**–**d** with N-alkyl-7-nitroindazoles **32a**–**b** to produce quinone oximes **33aa**–**db**.

In 2015, Kouakou et al. [59] used the already known approach of regioselective nucleophilic substitution of various 4-substituted benzyl cyanides **1a**–**d** with N-alkyl-7-nitroindazoles **32a**–**b** (Scheme 12). Reactions took place in methanolic solution of potassium hydroxide, forming corresponding 2-(7-hydroxyimino-1-alkyl-1,7-dihydroindazol-4-ylidene)-2-arylacetonitriles **33aa**–**db** (Table 2).

Alternative approaches to the synthesis of quinone methide oxime derivatives are severely underrepresented in the literature.

### 2.2. The Conversion of Quinone Methide Derivatives Using Hydroxylamine

As an alternative approach to the synthesis of quinone methide derivatives of oximes, the reaction of the corresponding quinone methide derivatives with the addition of hydroxylamine should be considered. This method has been known for a long time and has become more common in recent years for the conversion of a keto group into an oxime [87,88,89,90]. As an alternative transformation, which was not used for the synthesis of phenylcarboxymethylomethylenecyclohexa-2,5-dien-1-one oxime core, was presented by Wróbel in 2000 [80]. In that approach, the reaction between the nitroarene and phenylacetic ester furnished the nitroso compound which was in equilibrium with the oxime form.

**Scheme 13:**
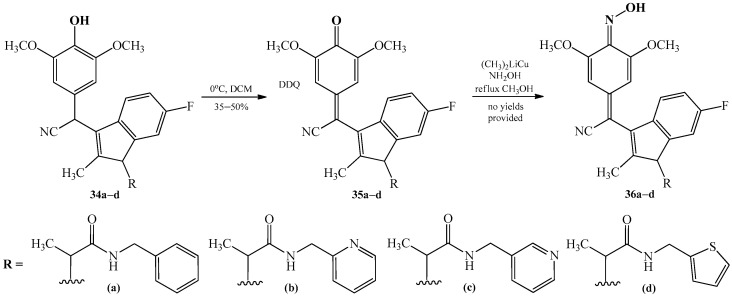
Two-stepped transformation reaction of hydroxylamine **34a**–**d** to produce quinone methide oximes **36a**–**d.** The structures of substituents R represent drawings (**a**–**d**).

In 2017, Piazza et al. [40] patented the synthesis of quinone methide oximes **36a**–**d**. The processes were two-stepped. The first stage consisted of conversion of the corresponding hydroxyl derivatives **34a**–**d** to quinone methides **35a**–**d**. The reactions were realized using 2,3-dichloro-5,6-dicyano-1,4-benzoquinone (DDQ). Dichloromethane (DCM) was used as a solvent. The reactions took place at 0 °C. As the last step, the quinone methides **35a**–**d** were converted to quinone methide oximes **36a**–**d** by the Gilman reagent ((CH_3_)_2_CuLi) and hydroxylamine (NH_2_OH), under reflux, with methanol as a solvent (Scheme 13).

**Scheme 14:**
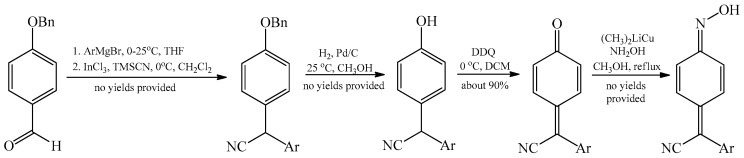
A concept of multistep transformation of 4-alkoxybenzaldehydes to produce arylcyanomethylene quinone oximes.

Methods applying quinone methides in obtaining arylcyanomethylenequinone oximes are virtually absent. This is due to the multitude of synthesis steps required in those type of processes leading to arylcyanomethylenequinone [91,92] (Scheme 14). In the presence of one-step condensation reaction of benzyl cyanide derivatives with 4-unsubstituted nitroarenes or their analogues, which is easier to adapt, the absence of processes deploying quinone methides is understandable.

## 3. Chemical Transformation of Arylcyanomethylenequinone Oximes

In the literature, chemical transformations of arylcyanomethylenequinone oximes are mostly represented by the conversion of oxime moiety and/or cyano group. From an oxime fragment, it is possible to obtain (a) the nitroso group by the isomerisation of quinone moiety; (b) amine by a reduction reaction; and (c) nitro compounds in an oxidation reaction. Under more “aggressive” reaction conditions, the conversion of the cyano group to (d) amine in a reduction reaction, and (e) eliminating of the cyano group in oxidative condition to form ketone, can also occur.

### 3.1. The Oxime–Nitroso Tautomerism Equilibrium in Arylcyanomethylenequinone Oxime

**Scheme 15:**
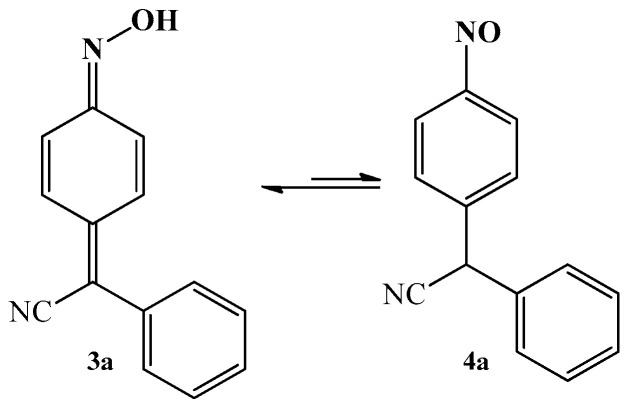
Oxime-nitroso tautomerism equilibrium between 4-(phenylcyanomethylene)-cyclohexa-2,5-dien-1-one oxime **3a** and 4-nitrosodiphenylacetonitrile **4a**.

The first aspect worth noting of the chemical transformations is the analysis of the arylcyanomethylenequinone oxime’s structure. During the analysis of the literature data, the question naturally arose regarding the possibility of oxime–nitroso tautomerism of these compounds. The opinions of various scientific researchers engaged in the study of the field turned out to be ambiguous. Thus, in works of different times, such as Davis et al. in 1960 [45], Zsako et al. in 1971 [93], Adam et al. in 2000 [94], Suwiński et al. in 2003 [81], Long et al. in 2013 [95], Hong et al. in 2015 [33], Kouakou et al. in 2015 [59], and Eddahmi et al. in 2019 [96], the authors exclude the possibility of existence of oxime-nitroso tautomerism in the arylcyanomethylenequinone oximes. On the other hand, some authors of recent decades allow the existence of oxime–nitroso tautomerism in these compounds. In 2010, Orlov et al. [86] showed a shift in the equilibrium of nitroso–oxime tautomerism towards the formation of the 4-(phenylcyanomethylene)-cyclohexa-2,5-dien-1-one oxime **3a** form (Scheme 15).

**Figure 3:**
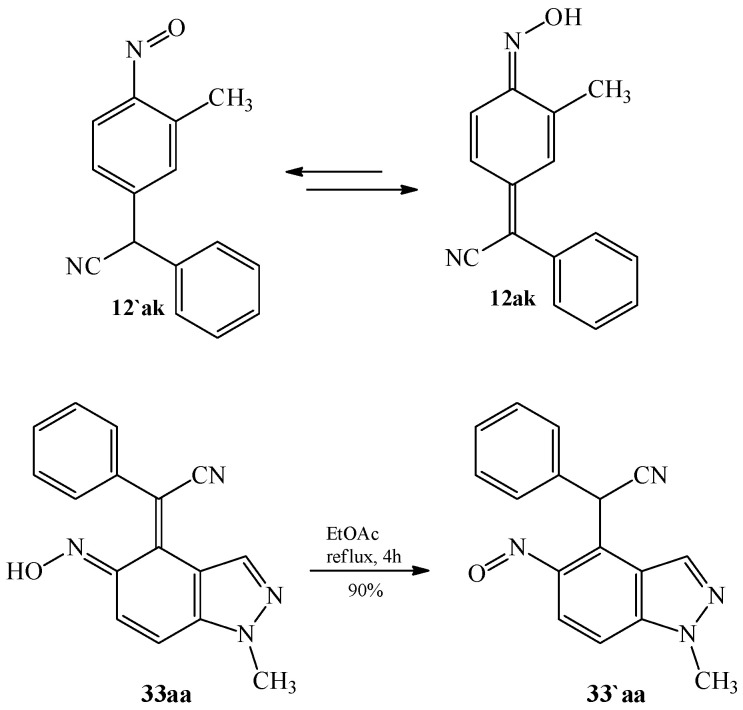
The structures of oximes **12ak**, **33aa,** and nitroso compound **33′aa**.

In turn, in 2000, Wróbel [80] noted the possibility of the existence of tautomeric nitroso-derivatives. In 2016, Hong et al. [60] studied the structure of 4-(phenylcyanomethylene)-2-methylcyclohexa-2,5-dien-1-one oxime **12ak** (Figure 3). In their work, they showed that the compound **12ak** can isomerize from the oxime to the corresponding nitroso-tautomer **12′ak** in solution, at different temperatures. Simultaneously, Hong et al. noted that the oxime form is the more stable one. Some other bicyclic particularly ortho-quinone metides derivatives, e.g., **33aa** proved to be existing in both forms and more stable, the nitroso **33′aa** form is formed during the heating of oxime [44].

**Figure 4:**
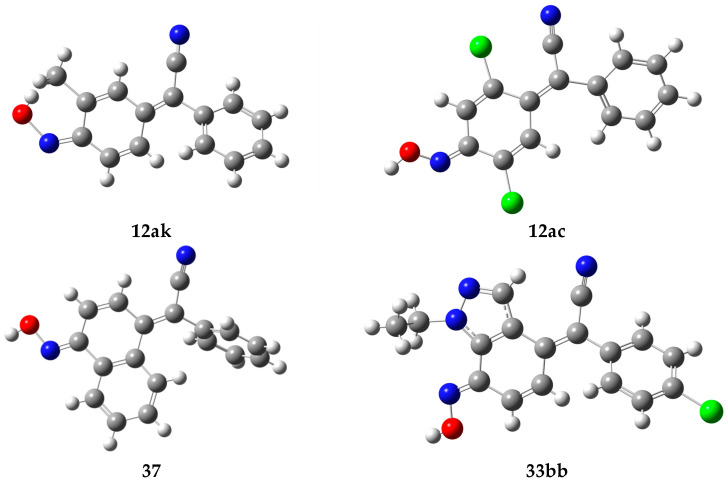
Structures of arylcyanomethylenequinone oximes confirmed by X-ray structural analysis. X-ray structures were extracted from: [60] for **12ak**, [33] for **12ac**, [81] for **37**, [59] for **33bb**.

The questions of the existence of oxime–nitroso tautomerism can be resolved by X-ray structural analysis. At the moment, among the arylcyanomethylenequinone oximes, four structures have been found using X-ray structural analysis (Figure 4). The structure proved high stability of para-quinone methide moiety in differently substituted and poly(hetero)cyclic arylcyanomethylenequinone oximes [33,59,60,81].

### 3.2. The Reduction Reactions of Arylcyanomethylenequinone Oxime

**Scheme 16:**
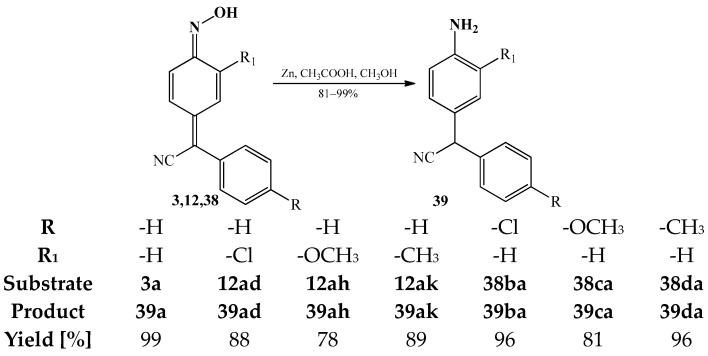
Reduction reaction of 4-(phenylcyanomethylene)-cyclohexa-2,5-dien-1-one oxime **3a** and its analogues **12ad**–**12ak** and **38ba**–**38da** to 4-(arylcyanomethyl)-arylamine **39a** and its analogues **39ad**–**39da**.

Mild chemical and catalytic reduction conversion of arylcyanomethylenequinone oximes to 4-aminoarylacetonitrile analogues has been the subject of several studies. An example of this reduction process is the reaction of 4-(phenylcyanomethylene)-cyclohexa-2,5-dien-1-one oxime **3a** and its analogues **12ad**–**12ak** and **38ba**–**da**. In the effect, the 4-(arylcyanomethyl)-arylamines **39a** and **39ad**–**39da** were synthesized [54,55,56,80,97]. The reactions were realized using zinc and acetic acid in methanolic solution (Scheme 16).

**Scheme 17:**
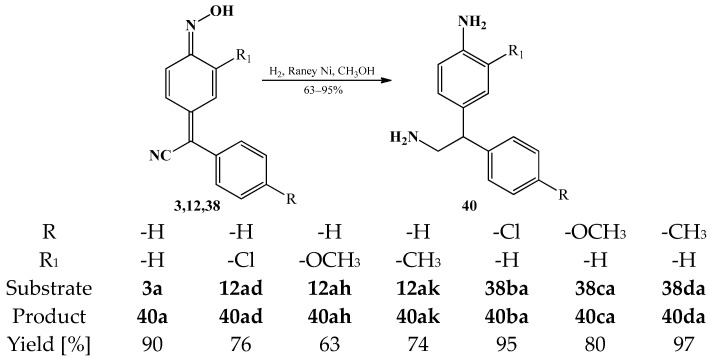
Reduction reaction of 4-(phenylcyanomethylene)-cyclohexa-2,5-dien-1-one oxime **3a** and its analogues **12ad**–**12ak** and **38ba**–**38da** to produce 1-amino-2-(4-aminophenyl)-2-phenyl-ethane **40a** and its analogues **40ad**–**40da**.

A more violent catalytic reduction of arylcyanomethylenequinone oximes converted both oxime moiety and cyano group. As a result, the corresponding 4-arylaminoarylethylamines are produced. Thanks to this the conversion of 4-(phenylcyanomethylene)-cyclohexa-2,5-dien-1-one oxime **3a** and its analogues **12ad**–**ak** and **38ba**–**da** to 1-amino-2-(4-aminoaryl)-2-aryl-ethanes **40a**, and **40ba**–**da** occurred [55,97]. The presented reduction reactions employ hydrogen gas and Raney nickel in methanolic solution (Scheme 17).

### 3.3. The Oxidation Reactions of Arylcyanomethylenequinone Oxime

**Scheme 18:**
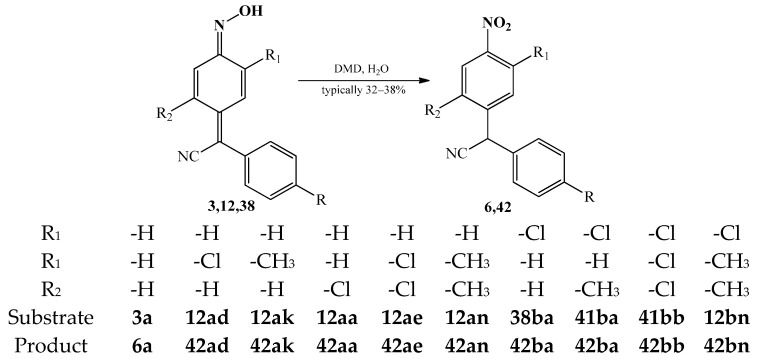
Oxidation reaction of 4-(phenylcyanomethylene)-cyclohexa-2,5-dien-1-one oxime **3a** and its analogues **12aa**–**12bn**, **38ba,** and **41ba**–**bb** to produce 4-(phenylcyanomethyl)-nitrobenzene **6a** and its analogues **42aa**–**bn**.

The oxidation process of arylcyanomethylenequinone oximes, also depending on the conditions of the reaction, can take place either only by the oxime group with the formation of corresponding nitro compounds or affecting both oxime and cyano moieties [57,58,85,94]. An example of the first oxidation process is the reaction of 4-(phenylcyanomethylene)-cyclohexa-2,5-dien-1-one oxime **3a** and its analogues **12aa**–**12bn**, **38ba** and **41ba**–**bb** producing 4-(phenylcyanomethyl)-nitrobenzene **6a** and its analogues **42aa**–**bn** [58,94]. The reactions were realized using dimethyldioxirane (DMD) as an oxidant and water as a solvent (Scheme 18). The products were obtained in low yields typically in a range of 32–38%.

**Scheme 19:**
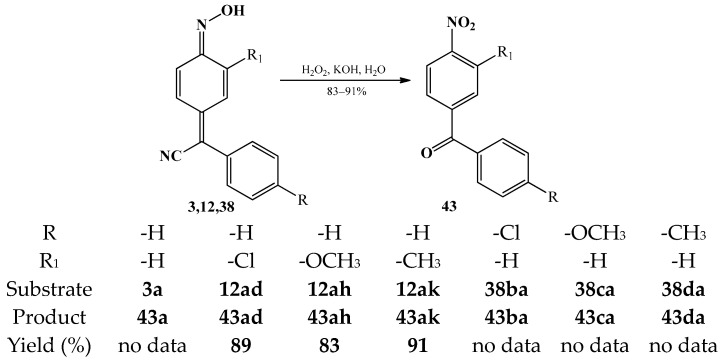
Oxidation reaction of 4-(phenylcyanomethylene)-cyclohexa-2,5-dien-1-one oxime **3a** and its analogues **12ad**–**ak** and **38ba**–**da** to produce (4-nitrophenyl)(phenyl)methanone derivatives **43a**–**bn**.

Simultaneous oxidative conversion of oxime moiety and the cyano group is possible using stronger oxidating agents. As a result, the corresponding (4-nitroaryl) (aryl) methanones are obtained, so that the conversion of 4-(phenylcyanomethylene)-cyclohexa-2,5-dien-1-one oxime **3a** and its analogues **12ad**–**ak** and **38ba**–**da** leads to (4-nitrophenyl)(phenyl)methanone derivatives **43a**–**bn** [57,85]. The oxidation processes are realized by treating the starting oximes **3a**, **12ad**–**ak,** and **38ba**–**da** with hydrogen peroxide in a boiling potassium hydroxide solution in a methanol/water mixture (Scheme 19).

### 3.4. The Substitution of a Hydrogen Atom at the Oxime Group in Arylcyanomethylenequinone Oxime

The chemical transformations of arylcyanomethylenequinone oximes by substitution of a hydrogen atom at the oxime group are represented by: acylations, alkylations or interaction of hydrogen with other groups, e.g., using sulfonyl chlorides or phosphoryl chlorides [23,24,25,32,42,59,97].

#### 3.4.1. The Acylation of a Hydrogen Atom at the Oxime Group

**Scheme 20:**
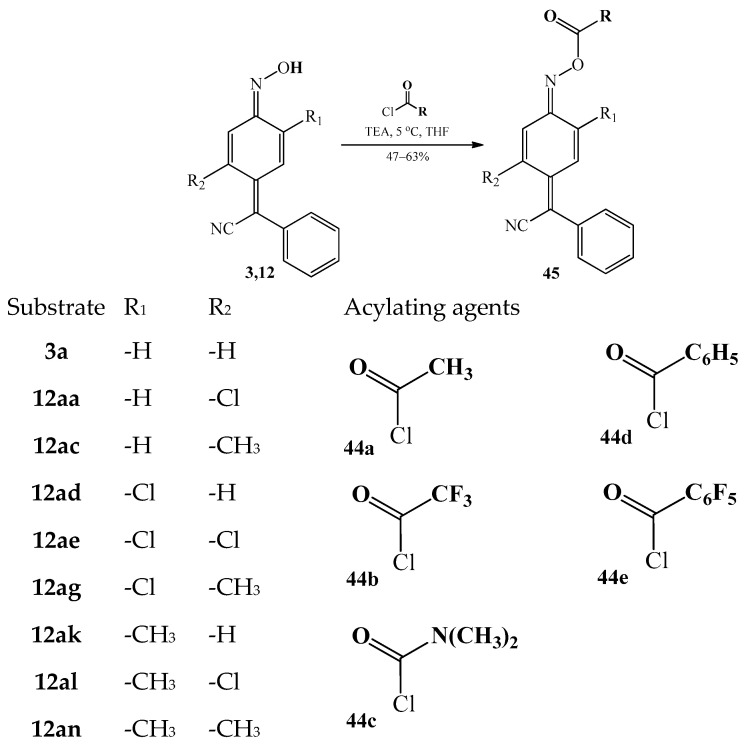
Acylation reaction of 4-(phenylcyanomethylene)-cyclohexa-2,5-dien-1-one oxime **3a** and its analogues **12aa**–**an**.

Several examples of acylation of 4-(phenylcyanomethylene)-cyclohexa-2,5-dien-1-one oxime **3a** and its analogues **12aa**–**an** are known [24,32,97]. Among others, acetyl chloride **44a**, trichloroacetyl chloride **44b**, 2-(dimethylamino)acetyl chloride **44c**, benzoyl chloride **44d** and pentafluorobenzoyl chloride **44e** are described, being used as acylation agents. The processes could be carried out in tetrahydrofuran (THF) with the addition of triethylamine (TEA) under cooling (Scheme 20). As a result of the reactions, nitroso derivatives **45a**_**a** to **45an**_**d** were obtained.

#### 3.4.2. The Alkylation of a Hydrogen Atom at the Oxime Group

**Scheme 21:**
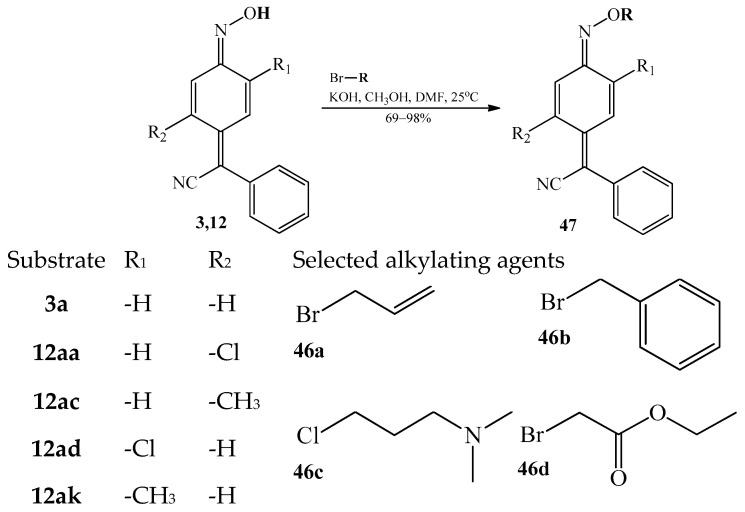
Alkylation reaction of 4-(phenylcyanomethylene)-cyclohexa-2,5-dien-1-one oxime **3a** and its analogues **12aa**–**ak**.

In a similar way to the acylation processes, the alkylation reactions of 4-(phenylcyanomethylene)-cyclohexa-2,5-dien-1-one oxime **3a** and its analogues 1**2aa**–**ak** are described [42]. Among others, allyl bromide **46a**, benzyl bromide **46b**, *N,N*-dimethylaminopropyl chloride **46c,** as well as ethyl bromoacetate 46d are used as alkylating agents. The reactions were carried out in dimethylformamide (DMF) with the addition of a methanolic solution of potassium hydroxide at the room temperature (Scheme 21). As a result of the reactions, *O*-alkylated oximes **47a**_**a**–**47ak**_**d** were obtained in typically high yields.

#### 3.4.3. Other Substitution Reactions at Oxygen Atom of the Oxime Group

**Scheme 22:**
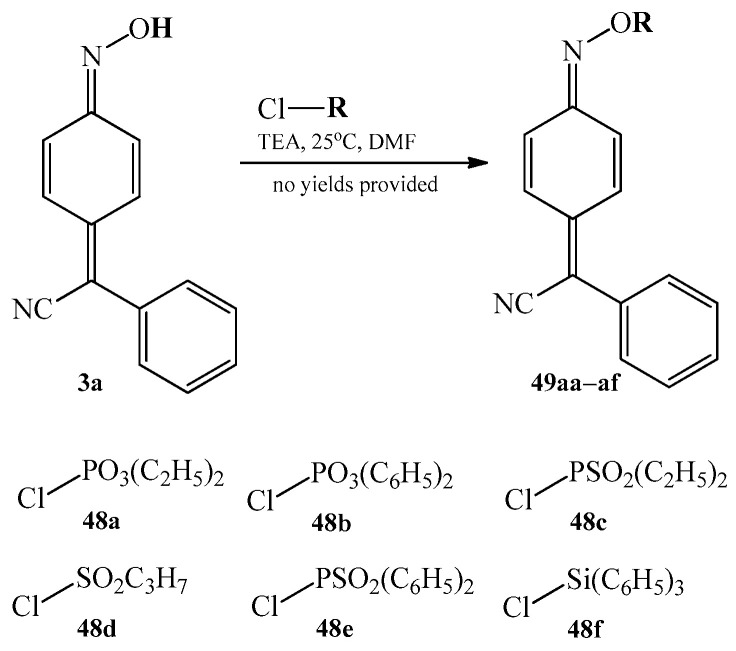
Summary of the substitution reactions of 4-(phenylcyanomethylene)-cyclohexa-2,5-dien-1-one oxime **3a** for phosphorus containing moiety.

In the literature, several examples of substitution reaction of the hydrogen atom of 4 arylcyanomethylenequinone oxime for phosphorus containing moiety are also reported. The first example includes a reaction series of 4-(phenylcyanomethylene)-cyclohexa-2,5-dien-1-one oxime **3a** with chlorides such as sulfonyl or phosphoryl chlorides as well as chlorosilane [24,25]. The reactions took place in the presence of triethylamine (TEA) at the room temperature and in dimethylformamide (DMF) as a solvent. Scheme 22 summarizes substitution agents, as well as possible products **49aa**–**af**, for these transformations.

**Scheme 23:**
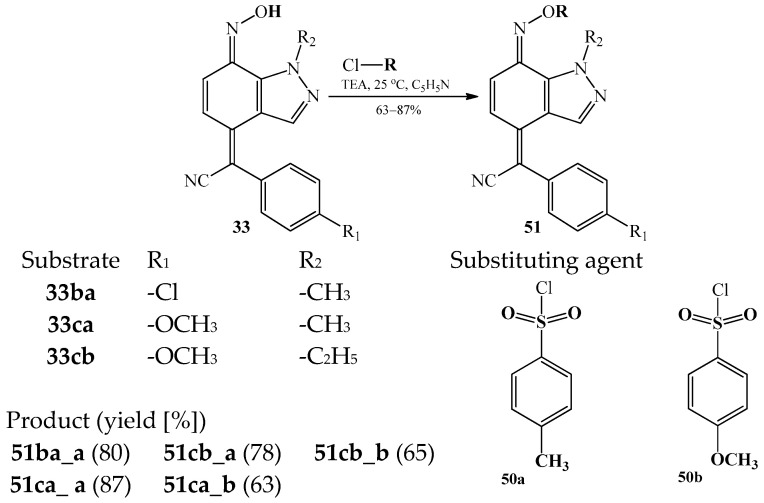
Summary of the substitution reactions of 2-(7-hydroxyimino-1-alkyl-1,7-dihydroindazol-4-ylidene)-2-arylacetonitriles **33aa**–**db** with 4-methylbenzylsulfonyl chloride **50a** or 4-methoxybenzylsulfonyl chloride **50b**.

Another example of substitution of the hydrogen atom in an oxime group is a series of reactions of 2-(7-hydroxyimino-1-alkyl-1,7-dihydroindazol-4-ylidene)-2-arylacetonitriles **33ba**, **33ca** and **33cb** with 4-methylbenzylsulfonyl chloride **50a** or 4-methoxybenzylsulfonyl chloride **50b** [23,59]. The reactions could be realized in the presence of triethylamine (TEA) at the room temperature and in pyridine solution. Scheme 23 summarizes possible products **51ba**, **51ca,** and **51cb** for these transformations [59].

## 4. Biological Properties of Arylcyanomethylenequinone Oximes

The creation of pharmaceuticals based on newly synthesized biologically active substances, in order to improve the medical provision of the population, is one of the main tasks of the healthcare system [98]. Thanks to the achievements of bioorganic chemistry, molecular biology (especially the determination of the structures of some receptors and enzymes by X-ray structural analysis), the structures of biological targets were identified, which led to the design of powerful and selective ligands or inhibitors. The synthesis of molecules acting on various biological targets and capable of modulating them has become an innovative approach to the design of drugs. An example of such an approach to designing new drugs can be the creation of “binary” structures containing several functionally significant parts of the molecule [99]. One of the promising classes of such compounds for the search for new biologically active substances could be arylcyanomethylenequinone oxime derivatives, which, on the one hand, contain several pharmacologically significant structure fragments in their structure, like oxime moiety, cyano group, quinonemethide fragment, and which still are small molecules, but on the other hand, which may not induct a “structural alerts” as less selective quinones [100]. It is not surprising that there are already numerous publications reporting on the various biological activities of arylcyanomethylenequinone oxime derivatives.

The first research references regarding biological activity of arylcyanomethylenequinone oximes were described in the middle of 1960s. At that time, Seidl [42] reported that the oximes proved to exhibit analgesic and antiallergic properties as well as these compounds can be useful against many strains of fungi, such as skin fungi and rotting fungi, and against parasites, such as trichomonas. In turn, in 1969 Zuccarello et al. [101] described potential of adrenal-inhibitory effects of arylcyanomethylenequinone oximes by inhibition of aldosterone biosynthesis. Some other types of biological activities of arylcyanomethylenequinone oximes were discovered in the end of the 1990s and more widely studied since then.

### 4.1. Antiallergic Activity

According to the literature, the arylcyanomethylenequinone oxime can be considered a promising antiallergic biologically active substance. In 1995, Gleason et al. [63] presented a study about the biological activity of arylcyanomethylenequinone oximes as a lipoxygenase inhibitor. The results show that oximes can inhibit both interleukin-4 (IL-4)-treated human monocyte 15-lipoxygenase (15-LO) and isolated soybean 15-lipoxygenase (15-LO) activity. In turn, in 1996, Freyne et al. [78] reported that arylcyanomethylenequinone oximes can also be potent inhibitors of both phosphodiesterase III and IV through downregulation of cells such as basophils and mast cells, which are useful not only in the treatment of allergic disorders, but also atopic diseases and related afflictions.

### 4.2. Antifungal Activity

In the two articles, Martyna et al. [62] in 2020 and Masłyk et al. [102] in 2019 reported biological activity of the simplest phenylcyanomethylenequinone oxime **3a**. The authors described that the 4-(phenylcyanomethylene)-cyclohexa-2,5-dien-1-one oxime **3a** acts as strongly antifungal against Candida, by inhibiting the formation of hyphae. Importantly, exploiting the path of reduction of the levels of cellular phosphoproteome, the compound **3a** did not show toxicity to human erythrocytes and zebrafish embryos. Additionally, the oxime **3a** effectively destroyed clinical isolates of Candida, affecting the mature biofilm, moderately disrupting membrane permeability and disrupting the growth of clinical isolates of fungi with a resistance to two systemic antifungal drugs—ketoconazole and caspofungin. Based on this, the authors proposed the mechanism of action of the agent 4-AN in *C. albicans* cells, which consists of the downregulation of cellular phosphorylation due to the inhibition of the activity of some protein kinases, which leads to the death of *C. albicans* cells mainly by necrosis.

### 4.3. Anti-Inflammatory Activity

**Figure 5:**
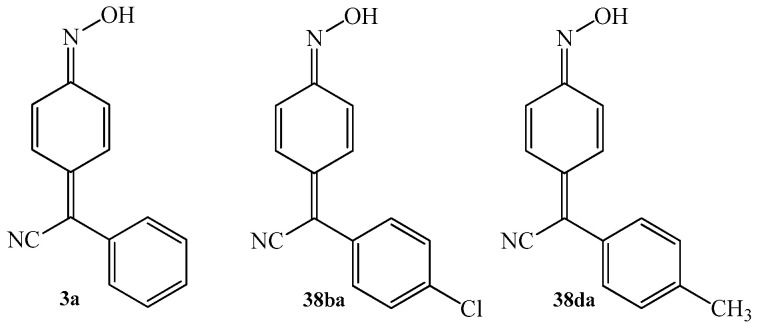
The chemical structure of 4-(phenylcyanomethylene)-cyclohexa-2,5-dien-1-one oxime **3a** and its derivatives **38ba**, **38da** with potential anti- inflammatory activity.

In 1984, Michel et al. [64] reported on the anti-inflammatory activity of arylcyanomethylene quinone oximes. That is related with the fact that these compounds can inhibit prostaglandin synthetase (PGS). This activity was tested for 4-(phenylcyanomethylene)-cyclohexa-2,5-dien-1-one oxime **3a**, 4-(4-chlorophenylcyanomethylene)-cyclohexa-2,5-dien-1-one oxime **38ba** and 4-(4-methylphenylcyanomethylene)-cyclohexa-2,5-dien-1-one oxime **38da** (Figure 5).

### 4.4. Anticancer Activity

**Figure 6:**
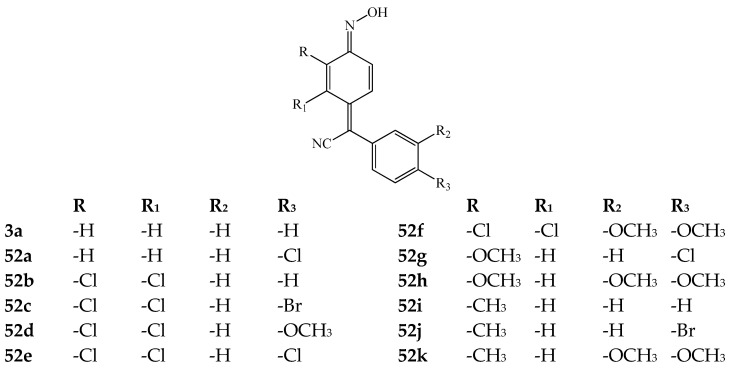
The chemical structure of 4-(phenylcyanomethylene)-cyclohexa-2,5-dien-1-one oxime **3a** and its derivatives **52a**–**k** with potential anticancer activity.

Many articles [39,40,43,60] describe the anticancer activity of arylcyanomethylene quinone oximes and their derivatives. According to the literature, the first information about this kind of research was published in 1975 by Dore and Viel [43]. In 1990, Freyne et al. [39] tested and described 4-(phenylcyanomethylene)-cyclohexa-2,5-dien-1-one oxime analogues. The authors noted that the studied compounds inhibit the elimination of retinoic acids from blood plasma. Those compounds can be used in the treatment of disorders characterized by increased proliferation and/or abnormal differentiation of epithelial cells. In turn, Hong et al. [60] synthesized and tested several 4-(phenylcyanomethylene)-cyclohexa-2,5-dien-1-one oxime **3a** and their derivatives **52a**–**k** (Figure 6). The authors reported that compounds **3a** and **52a**–**k** displayed strong antitumor activity against Caki-1 and 769-P.

**Figure 7:**
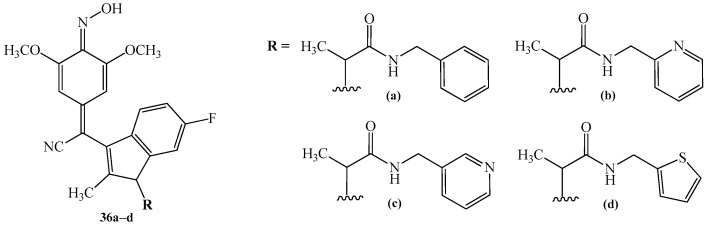
The chemical structure of oximes **36a**–**d** with potential anticancer activity (**a**–**d**).

In 2017, Piazza et al. [40] carried out biological studies of patented oximes **36a**–**d** (Figure 7). The aim of these studies was to establish an orthotopic mouse model of lung cancer using A549 human lung adenocarcinoma cells, and to investigate the therapeutic efficacy of oximes **36a**–**d**. Based on obtained results, the authors stated that compounds **36a**–**d** are promising anticancer agents as RAS inhibitors. The human cancer cells used in this research included A-549, HT-29, MDA-MB-231, Colo-205, Caco2, HCT-116, SW-480, and DLD-1.

### 4.5. Positive Inotropic and Lusitropic Effects

In their reports, Freyne and Raeymaekers [77,103] described positive inotropic and lusitropic effects of arylcyanomethylenequinone oximes. The authors showed that these compounds can be potent inhibitors of the phosphodiesterase type IIIc (cardiotonic-sensitive PDE III). Inhibition of PDE IIIc leads to an elevation of cAMP in cardiac muscle, which in turn enhances sarcolemmal entry of Ca^2^⁺ into the cell, increases the release and reuptake of Ca^2^⁺ by the sarcoplasmic reticulum, and probably also increases the sensitivity of contractile proteins to Ca^2^⁺. As a result, an increased contractile force of the heart ensues (positive inotropy) as well as a faster relaxation of the heart (positive lusitropy). Particularly important is the observation that the positive inotropic and lusitropic effects generally do not coincide with a simultaneous increase of other haemodynamic variables such as heart rate and blood pressure. Concomitant increases of heart rate and/or blood pressure would indeed put an extra strain on the heart and cancel the beneficial positive cardiac inotropy and lusitropy.

In vivo experiments with these oximes show moderate systemic vasodilation and hence, a decrease in blood pressure. The heart rate generally only increases at high doses. Overall, arylcyanomethylenequinone oximes caused a dramatic increase in cardiac output by cardiac positive inotropy and lusitropy and without a major influence on heart rate and/or blood pressure. Consequently, the oximes can be considered to be therapeutical drugs for the treatment of those suffering from Congestive Heart Failure. Said condition may result from a heart attack, infection of the heart, chronic hypertension, deficiencies in the operation of the heart valves and other disorders of the heart, leading to congestive heart failure (CHF).

### 4.6. Potential Application of Arylcyanomethylenequinone Oximes in Veterinary Medicine

**Figure 8:**
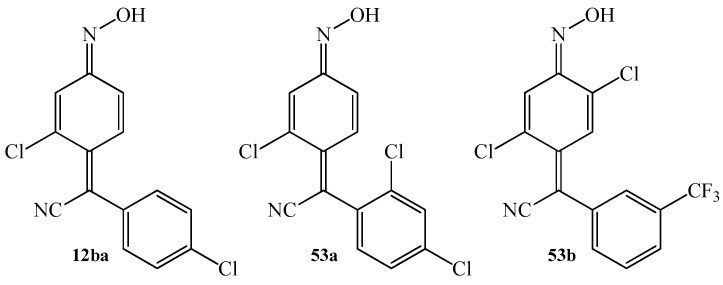
The chemical structure of oximes **12ba** and **53a**–**b** with potential anthelmintic and coccidiostatic activity.

The application of arylcyanomethylenequinone oximes in veterinary medicine have also been described several times in the literature. These compounds can be successfully applied as anthelmintics and coccidiostats. The most important compounds that show these properties include: 4-(4-cholophenylcyanomethylene)-3-chlorocyclohexa-2,5-dien-1-one oxime **12ba**, 4-(2,4-dicholophenylcyanomethylene)-3-chlorocyclohexa-2,5-dien-1-one oxime **53a,** and 4-(3-trifluoromethylphenylcyanomethylene)-2,5-dichlorocyclohexa-2,5-dien-1-one oxime **53b** (Figure 8). The compounds **12ba**, **53a**–**b** were the subject of tests on animals. In 1973, Anssen et al. [61] reported about the application of oximes **12ba**, **53a**–**b** as feed additives for chickens against Eimeria. According to the reports, simultaneously, mortality decreases and the recovery of animals can be observed. In turn, in 1976, Janssen and Sipido [104] presented very high potency of oximes **12ba**, **53a**–**b** against the large American liver fluke in sheep.

**Figure 9:**
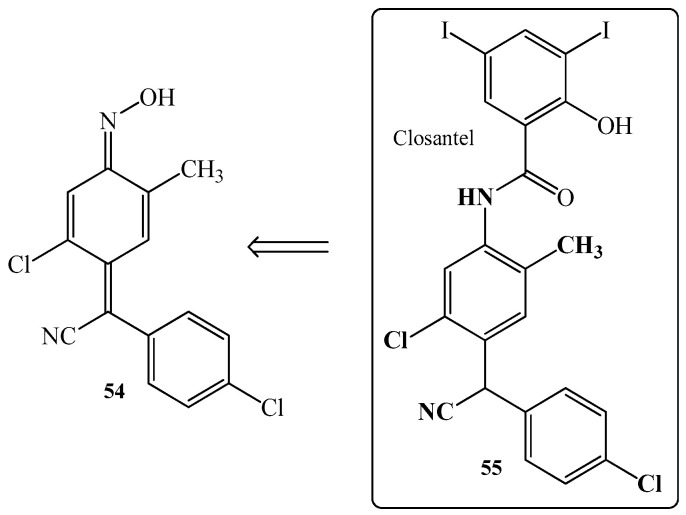
The chemical structure of 4-(4-cholophenylcyanomethylene)-2-methyl-5-chlorocyclohexa-2,5-dien-1-one oxime **54** and medicine Closantel **55**.

Another interesting compound related with veterinary medicine is 4-(4-cholophenylcyanomethylene)-2-methyl-5-chlorocyclohexa-2,5-dien-1-one oxime **54** (Figure 9) [56,105,106,107,108]. The modification of oxime **54** allows for the synthesis of the drug Closantel **55** (Figure 9) which is a halogenated salicylanilide with potent anti-parasitic activity. It is widely used in the management of parasitic infestation in animals but is contraindicated in humans. In 2016, Tabatabaei et al. [109] reported a patient example of unintentional ingestion of Closantel (**55**). After application of the drug, the symptoms such as the progressive loss of vision in both eyes and mild headache were observed. The research results showed destruction in the outer retina according to Optical Coherence Tomography (OTC) and possibly, visual pathways according to visual evoked potential (VEP) and electroretinogram (ERG), and confirmed toxic optic neuropathy. The brain imaging was normal. In turn, mild anaemia and liver transaminase enzymes elevation were the only systemic complications.

### 4.7. Potential Application of Arylcyanomethylenequinone Oximes as Plant Protection Products

**Figure 10:**
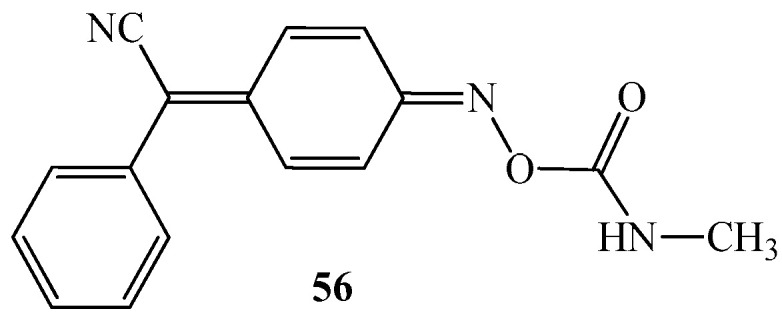
The chemical structure of 4-(α-cyanobenzylidene)-2,5-cyclohexadiene-4-one *O-*(methylcarbamoyl) oximes **56** as potential herbicide agent.

In several documents, it is mentioned that arylcyanomethylenequinone oximes can be used as plant protection products, e.g., herbicides [32,65] and fungicides [66]. One of the most important and studied representatives of herbicides is 4-(α-cyanobenzylidene)-2,5-cyclohexadiene-4-one *O-*(methylcarbamoyl) oximes **56** (Figure 10). The compound **56** was tested in 1973 by Dixon [32]. The author reported that oxime **56** next to the destroying properties of selective plants can also stimulate plant growth as well as serve as inhibitor of the enzymatic activity of certain insects.

**Figure 11:**
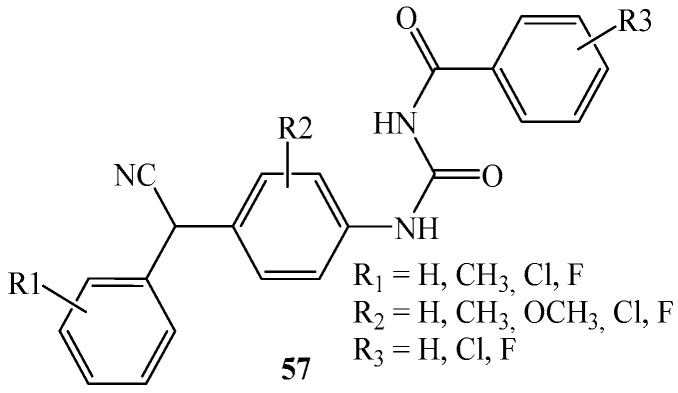
The general structure of **57**.

In 1986, D’Silva [67] reported about a series of ureas (**57**) derived from the corresponding arylcyanomethylenequinone oximes. D’Silva claimed that the ureas can be successfully used as pesticide agents (Figure 11).

According to the author, compounds **57** prevent attack by insects and they have relatively high residual toxicity. In turn, with respect to plants, they have a high margin of safety in that when used in a sufficient amount to kill or repel the pests, they do not burn or injure the plant, and they resist weathering that includes wash-off caused by rain, decomposition by ultraviolet light, oxidation, or hydrolysis in the presence of moisture. In addition, these oximes may be used in the soil, upon the seeds, or the roots of plants without injuring either the seeds or roots of plants. In the patent [67], the tests on larvae of the southern armyworm, the Mexican bean beetle, the tobacco budworm, and the cotton bollworm were described. In all cases, the mortality of larvae was recorded, caused by arylcyanomethylenequinone oximes urea.

## 5. Conclusions and Future Outlook

As can be deduced from the above analysis of the literature, the presented approach to the synthesis of arylcyanomethylenequinone oximes is characterized by relative easiness of preparation (it is not multi-staged, nor does it require the use of oxidizing agents that can lead to the formation of by-products and isomeric mixtures, and the use of catalysts and especially prepared solvents is also unnecessary). Condensation reactions of (hetero) arylacetonitriles with (hetero)nitroarenes are characterized by an employment of easily available starting reagents, regioselectivity, and simplicity. They present a facile way to synthesize not only mono-, but also bis-derivatives of arylcyanomethylenequinone oximes or their heterocyclic analogues.

Analysis of the literature also clearly showed that arylcyanomethylenequinone oxime derivatives are versatile feedstock to production of their nitro, keto, and amino derivatives. What is even more important, the chemical transformations of said oximes occur in mild conditions, simplifying preparation of various systems containing aryl-groups.

The wide palette of biological activity, e.g., cardiotonic-sensitive PDE III inhibitory, kinase inhibitory, RAS inhibitory, anticancer, anti-inflammatory, antiallergic, antifungal, anti-parasitic, as well as plant protection activity, e.g., herbicidic, fungicidic, and pesticidic activity, are reported to be found in arylcyanomethylenequinone oximes. Further studies of the bioactivity of the discussed group of compounds lead to uncovering of unexpected diversity of various biological activities, useful from the standpoint of industrial and medical applications.

Despite certain progress made in chemical and biological studies of arylcyanomethylenequinone oximes, the application potential of them is still underestimated. This could be rationalized by taking into consideration the fact that the mode of their action and biochemical mechanisms are still waiting to be discovered to give a rise to the new drugs based on their core.

To summarize, arylcyanomethylenequinone oxime derivatives deserve further studies, both in the fields of their transformations and their potential application in medicine, agriculture, and industry, as they present a promising source of innovation in those fields.

## Figures and Tables

**Table 1:** Analogues of arylcyanomethylenequinone oximes **12aa**–**cn**.

Product (Yield [%])		R	R_1_	R_2_	R_3_	Product (Yield [%])		R	R_1_	R_2_	R_3_
**12aa**	(53)	-H	-H	-H	-Cl	**12be**	(94)	-Cl	-Cl	-H	-Cl
**12ab**	(25)	-H	-H	-H	-OCH_3_	**12bf**	(92)	-Cl	-Cl	-Cl	-H
**12ac**	(76)	-H	-H	-H	-CH_3_	**12bg**	(77)	-Cl	-Cl	-H	-CH_3_
**12ad**	(92)	-H	-Cl	-H	-H	**12bh**	(80)	-Cl	-OCH_3_	-H	-H
**12ae**	(93)	-H	-Cl	-H	-Cl	**12bi**	(81)	-Cl	-OCH_3_	-H	-Cl
**12af**	(53)	-H	-Cl	-Cl	-H	**12bj**	(80)	-Cl	-OCH_3_	-H	-OCH_3_
**12ag**	(77)	-H	-Cl	-H	-CH_3_	**12bk**	(60)	-Cl	-CH_3_	-H	-H
**12ah**	(87)	-H	-OCH_3_	-H	-H	**12bm**	(80)	-Cl	-CH_3_	-Cl	-H
**12ai**	(82)	-H	-OCH_3_	-H	-Cl	**12bn**	(43)	-Cl	-CH_3_	-H	-CH_3_
**12aj**	(88)	-H	-OCH_3_	-H	-OCH_3_	**12ce**	(91)	-OCH_3_	-Cl	-H	-Cl
**12ak**	(72)	-H	-CH_3_	-H	-H	**12cg**	(80)	-OCH_3_	-Cl	-H	-CH_3_
**12al**	(92)	-H	-CH_3_	-H	-Cl	**12cf**	(65)	-OCH_3_	-Cl	-Cl	-H
**12am**	(82)	-H	-CH_3_	-Cl	-H	**12ci**	(84)	-OCH_3_	-OCH_3_	-H	-Cl
**12an**	(53)	-H	-CH_3_	-H	-CH_3_	**12cj**	(65)	-OCH_3_	-OCH_3_	-H	-OCH_3_
**12ba**	(100)	-Cl	-H	-H	-Cl	**12cl**	(88)	-OCH_3_	-CH_3_	-H	-Cl
**12bb**	(89)	-Cl	-H	-H	-OCH_3_	**12cm**	(80)	-OCH_3_	-CH_3_	-Cl	-H
**12bd**	(100)	-Cl	-Cl	-H	-H	**12cn**	(69)	-OCH_3_	-CH_3_	-H	-CH_3_

**Table 2:** Analogues of 2-(7-hydroxyimino-1-alkyl-1,7-dihydroindazol-4-ylidene)-2-arylacetonitriles **33aa**–**db**.

Product (Yield (%))		R	R_1_	Product (Yield (%))		R	R_1_
**33aa**	(67)	-H	-CH_3_	**33ca**	(65)	-OCH_3_	-CH_3_
**33ab**	(69)	-H	-C_2_H_5_	**33cb**	(70)	-OCH_3_	-C_2_H_5_
**33ba**	(86)	-Cl	-CH_3_	**33da**	(90)	-CH_3_	-CH_3_
**33bb**	(76)	-Cl	-C_2_H_5_	**33db**	(92)	-CH_3_	-C_2_H_5_

## Data Availability

Not applicable.
